# Gait Impairment in a Rat Model of Focal Cerebral Ischemia

**DOI:** 10.1155/2013/410972

**Published:** 2013-03-03

**Authors:** Saara Parkkinen, Francisco J. Ortega, Kristina Kuptsova, Joanna Huttunen, Ina Tarkka, Jukka Jolkkonen

**Affiliations:** ^1^Department of Neurology, Institute of Clinical Medicine, University of Eastern Finland, Yliopistonranta 1 C, 70210 Kuopio, Finland; ^2^A. I. Virtanen Institute for Molecular Sciences, University of Eastern Finland, 70210 Kuopio, Finland; ^3^Department of Health Sciences, University of Jyväskylä, 40014 Jyväskylä, Finland

## Abstract

The availability of proper tests for gait evaluation following cerebral ischemia in rats has been limited. The automated, quantitative CatWalk system, which was initially designed to measure gait in models of spinal cord injury, neuropathic pain, and peripheral nerve injury, is said to be a useful tool for the study of motor impairment in stroke animals. Here we report our experiences of using CatWalk XT with rats subjected to transient middle cerebral artery occlusion (MCAO), during their six-week followup. Large corticostriatal infarct was confirmed by MRI in all MCAO rats, which was associated with severe sensorimotor impairment. In contrast, the gait impairment was at most mild, which is consistent with seemingly normal locomotion of MCAO rats. Many of the gait parameters were affected by body weight, walking speed, and motivation despite the use of a goal box. In addition, MCAO rats showed bilateral compensation, which was developed to stabilize proper locomotion. All of these interferences may confound the data interpretation. Taken together, the translational applicability of CatWalk XT in evaluating motor impairment and treatment efficacy remains to be limited at least in rats with severe corticostriatal infarct and loss of body weight.

## 1. Introduction

Stroke imposes an enormous economic and human burden. Despite some spontaneous recovery observed during the first 3 months, around half of stroke patients are left with permanent disability, in which upper extremity motor impairment is the most prominent. Most hemiplegic patients also have a gait abnormality including decreased velocity, cadence, stride length, and prolonged swing phase on the affected side [1]. In addition to reduced ambulation, this could impair balance and lead to falls [2]. 

Perhaps the most common experimental stroke model is transient middle cerebral artery occlusion (MCAO) [3], which reproduces many features of human stroke. Numerous tests are available to assess behavioral impairment in MCAO rats, varying from simple tasks measuring general severity of neurological impairment to more demanding reaching tasks that measure upper extremity function [3–5]. Versatile analysis of gait and ambulation has been limited in stroke animals until the CatWalk system was recently introduced as an automated and quantitative gait analysis tool. It is based on video analysis of light reflected by the paws as they contact the glass floor. This represents a rapid way to objectively quantify several gait parameters such as position, pressure and surface area of each paw, which are used to calculate spatial paw statistics, the relative positions between paws, temporal parameters of gait, and interlimb coordination. This system has clinical relevance because the principle is very similar to the GAITRite system that can assess gait in stroke patients [1].

Recently, four papers have described the use of CatWalk in experimental stroke models. Wang et al. [6] studied gait 4 days and 5 weeks after cortical lesion (pMCAO model). Four days after ischemia, the intensity and maximal area of the affected forepaw were significantly decreased. They also found impairment of interlimb coordination. Most of these impairments persisted for 5 weeks. Vandeputte et al. [7] showed reduced intensity, print area, and width max area of the contralateral hindlimbs one day after cortical photothrombosis (Rose Bengal model). Encarnacion et al. [8] tested two rat strains after transient MCAO (filament model). They showed short-term deficits in intensity, stride length, stand index, duty cycle, and placement time of the impaired forelimb. In this study, deficits in hindlimb swing speed and placement time were more long lasting. Hetze et al. [9] showed decreases in maximum contact area, stride length, and swing speed in the impaired hindlimb following transient MCAO in mice.

MCAO rats usually develop compensatory strategies to overcome motor deficits. This has not been evaluated in the aforementioned studies although there is data available for all limbs. Another issue that has not been discussed is the difficulty to motivate the rats to cross the runway in a consistent manner without stopping and turning around. To overcome this, a goal box can be mounted at the end of the runway in the CatWalk XT version 9.1. Here we report our experiences in using CatWalk XT with a goal box in rats subjected to transient MCAO.

## 2. Material and Methods 

### 2.1. Animals

Male Wistar rats (BE Harlan Laboratories Ltd., Israel), 3 months old, weighing 350–400 g at the beginning of the study were used. The rats were housed individually under 12 h/12 h day and night cycles in a temperature-controlled environment (20 ± 1°C). All animal procedures were approved by the Animal Ethics Committee (Hämeenlinna, Finland) and conducted in accordance with the guidelines set by the European Community Council Directives 86/609/EEC. All efforts were made to minimize the number of animals used and to ensure their welfare throughout. 

### 2.2. Middle Cerebral Artery Occlusion

Focal cerebral ischemia was induced by the intraluminal filament technique (*n* = 7) [10]. Under halothane anesthesia, the right common carotid artery was exposed through a midline cervical incision. A heparinized nylon filament (diameter 0.25 mm, rounded tip) was inserted into the stump of the external common carotid artery. The filament was advanced 1.8–2.1 cm into the internal common carotid artery until resistance was felt. After 60 minutes of occlusion, the filament was removed and the external carotid artery was permanently closed by electrocoagulation. The sham-operated rats (*n* = 6) were treated in a similar manner, except that the filament was not placed into the internal carotid artery. The neurological impairment was assessed 24 hours after MCAO using a modified version of the limb-placing test [11] and animals with no behavioral impairment were excluded from the study. 

### 2.3. Magnetic Resonance Imaging

Quantification of MR images was performed 24 hours after MCAO with a Bruker 7 T horizontal scanner to exclude the animals with no cortical damage or those with signs of hemorrhage. Based on MRI images, 3 animals were excluded from the study. For determination of the infarct volume, the rats were anesthetized with 5% isoflurane in a gas mixture of 30% O_2_/70% N_2_O. After induction, anesthesia was maintained throughout the imaging with 2.5% isoflurane inhaled through a nose mask. *T*_2_-weighted multislice images were acquired using a RARE sequence with the following parameters: time-to-repetition TR = 2.5 s, effective time-to-echo effTE = 40 ms, RARE factor 8, matrix size of 256 × 256, field-of-view of 30 mm × 30 mm, and BR 15 slices with a slice thickness of 1 mm. Infarct volumes were analyzed by using ImageJ. Areas of surviving gray matter in the cortex and striatum were outlined for each hemisphere. The difference between the size of an intact area in the contralateral hemisphere and the respective residual area in the ipsilateral hemisphere was recorded as the infarcted area. The total infarct volume was calculated by multiplying the infarct area by the distance between the slices and summing together the volumes. 

### 2.4. CatWalk Test

CatWalk XT 9 (Noldus, The Netherlands), a quantitative gait analysis system, was used for this study. An enclosed glass walkway is illuminated from the long edge with a green light that is completely internally reflected. The light reflected by the paws as they contact the glass floor is captured by a high-speed video camera, which is then transformed into a digital image. The walkway was fixed to 90 mm wide. The camera was positioned 40 cm below the walkway and automatic detection settings were applied. An intensity threshold was set to 0.11, the camera gain was set to 18, and the maximum allowed speed variation was set to 50%. The animals were trained for three days. On the first day of training the lights in the room were on and food pellets were placed in the goal box to motivate the animals to complete the task. On the second and third days, the training took place in the dark with the only light source coming from the computer screen, where the CatWalk system was activated, but the program was not used. Rats were subjected to gait assessment at days 0 (baseline), 6, 21, and 42 after MCAO or sham surgery. Tests were performed in the same conditions as the training sessions, with the only exception that another male Wistar rat (non-testing) was systematically put in the goal box to motivate the trial rats to run towards it. The same motivator rat was used for all animals and in all test sessions. In case the animal was not motivated by the goal box, alternative positive motivators were used such as noise and food reward. Otherwise, animals were allowed to run back and forth on the walkway until 4 accepted runs were collected. An observer blind to the experimental groups performed the behavioral analysis and the data analysis. 

During the data analysis the steps were automatically labeled as right fore paw (RF), right hind paw (RH), left fore paw (LF), and left hind paw (LH), in which the right stands for the nonimpaired side and the left for the impaired side. Faulty labels caused by tail, whiskers, or genitalia were removed. After identification of individual footprints, we performed an automated analysis of a wide range of parameters. Data were classified as follows: (1) individual paw statistics; (2) comparative paw statistics; (3) interlimb coordination; (4) temporal parameters. In addition to the automatic values we also analyzed a package of individual footprint parameters such as the paw angle, toe spread, print length, and intermediate toe spread (Table 1). One trial consisted of four runs and at least one successfully recorded print from each paw was counted in from each run. Toe spread was determined as the distance between the innermost and outermost toes of the foot. Intermediate toe spread in the four-toed forepaw print was the distance between the innermost toe and the toe next to the outermost toe. In the five-toed hindpaws the intermediate toe spread was determined as the distance between the second and the third toes. The paw angle was determined simultaneously with print length by drawing a line from the back of the palm and following the pads of the paw to the second toe from the centre. 

### 2.5. Statistics

All statistical tests were performed with GraphPad Prism5 statistical software (La Jolla, CA, USA). CatWalk data for the overall group effect and group × time interaction were analyzed using two-way repeated measures ANOVA followed by Bonferroni's post hoc comparison tests when appropriate. Linear correlations between body weight, speed, and gait parameters were evaluated by the Pearson's product-moment correlation coefficient. All values are presented as mean ± standard error of mean (SD). *P* values <0.05 were considered significant.

## 3. Results

### 3.1. Corticostriatal Infarction Affects Gait Bilaterally

**Figure 1:**
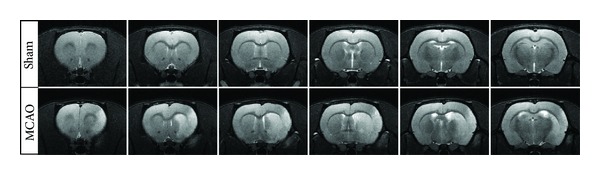
Corticostriatal lesion. Representative *T*_2_-weighted magnetic resonance images of coronal sections acquired 24 hours after MCAO.

Transient MCAO resulted in variable cortical infarction and included most of the parietal sensorimotor cortex. Typically the striatum was completely damaged (Figure 1). Quantification of MR images made 24 hours after surgery showed a severe corticostriatal infarct in all the included MCAO rats (cortex 77.7 ± 15.4; striatum 32.9 ± 0.55 mm^3^). This was associated with severe, long-lasting behavioral impairment compared to sham-operated rats at the end of the followup when assessed by the cylinder test (35.6 ± 4.7% reduction of the impaired forelimb use), sticky label test (719 ± 120% increase in time to remove the label), and Montoya's staircase (43.6 ± 3.2% reduction in the number of eaten pellets). 

**Figure 2:**
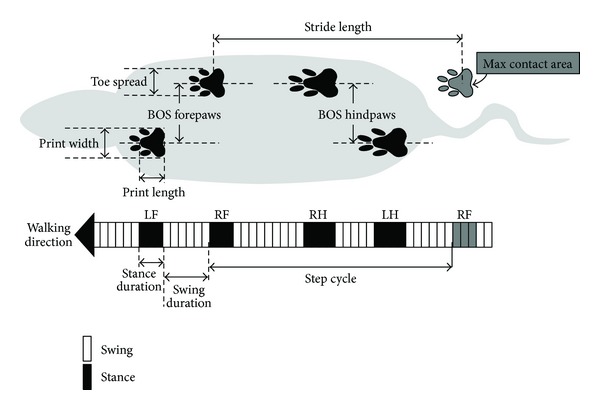
Graphical representation of selected gait parameters. The animal is walking towards the left. Black and white boxes represent time fractions where the paw is in contact with the surface or lifted at walking. RF: right forelimb; LF: left forelimb; RH: right hindlimb; LH: left hindlimb.

Gait parameters were first analyzed to compare impairment between the contralateral (impaired) versus ipsilateral (non-impaired) paws within groups (Figure 2). Prior to their operation, both sham-operated and MCAO rats showed no bias in their use of impaired and non-impaired fore or hindpaws. Interestingly, although MCAO animals displayed functional deficits in some of the CatWalk parameters, we did not find significant differences when comparing the contralateral and ipsilateral sides. 

### 3.2. Temporal Parameters Are Affected by MCAO

**Figure 3:**
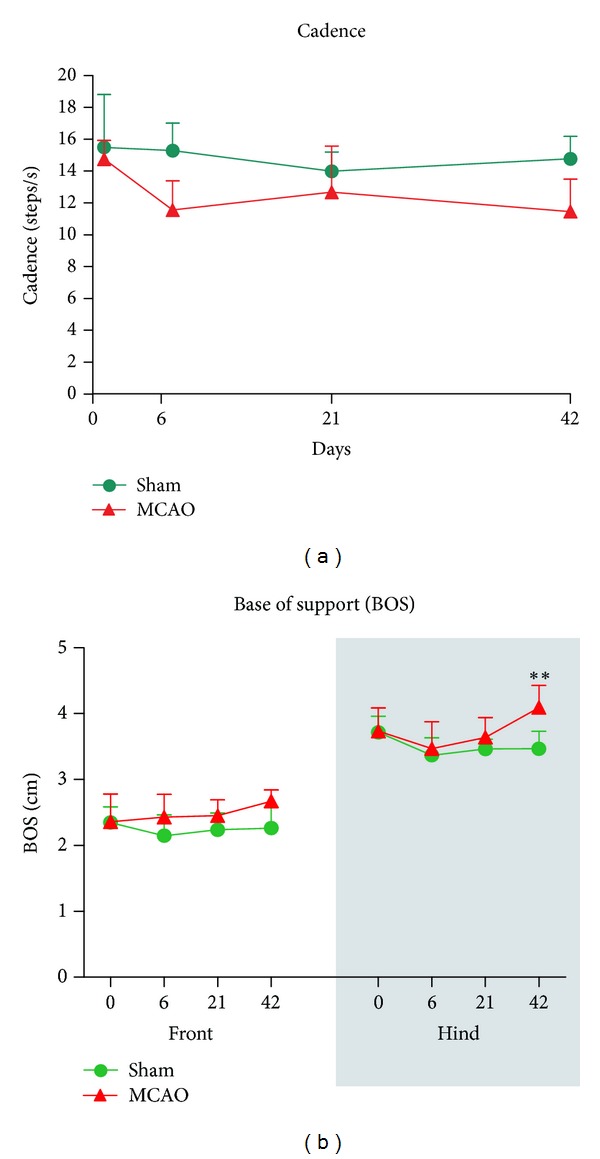
Focal cerebral ischemia significantly affected cadence and base of support (BOS). (a) MCAO animals showed a decreased number of steps per second (cadence) when walking along the CatWalk runway at postoperative days 6 and 42. (b) Ischemic animals showed significantly larger hindlimb BOS at postoperative day 42. All values are given as mean ± SD. Statistics: **P < 0.01 versus sham-operated group.

Cadence describes the number of steps per second that the animal makes along the walking path. Similar to that reported in stroke patients, MCAO rats showed significant decrease in cadence (Figure 3(a); group effect *F*_1,33_ = 9.611, *P* < 0.05; and time effect *F*_1,33_ = 3.173, *P* < 0.05). This parameter is directly affected by the total run duration and the speed of the animal. 

**Figure 4:**
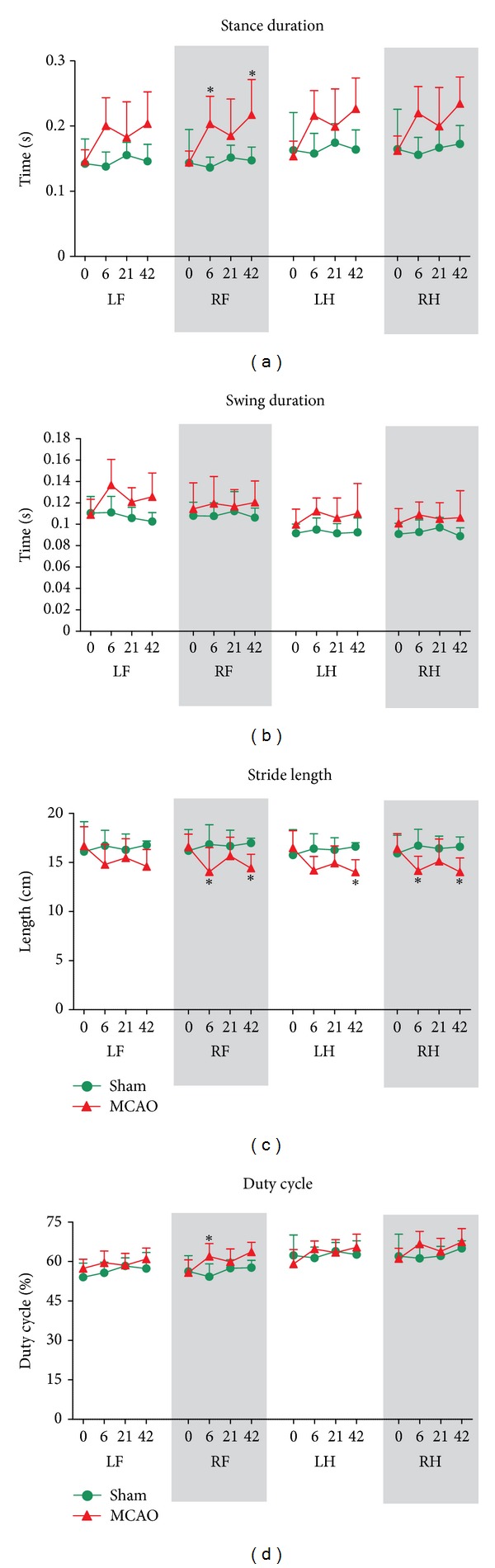
Effect of focal cerebral ischemia on temporal and comparative paw parameters. (a) Cerebral ischemia increased the duration of the stance phase of all paws at postoperative days 6 and 42. (b) Swing duration was only significantly different in the left forelimb (LF) at the acute phase after ischemia. (c) Stride length of MCAO animals was generally shorter. (d) Only the right forelimb (RF) of MCAO animals denoted longer duty cycle at postoperative day 6. All values are given as mean ± SD. Statistics: *P < 0.05 versus sham-operated group.

The stance duration (average time in seconds that the paw is in contact with the glass plate for each step) in MCAO rats was increased for the LF (group effect *F*_1,33_ = 6.95, *P* < 0.05), RF (group effect *F*_1,33_ = 8.69, *P* < 0.05, time effect *F*_1,33_ = 2.91, *P* < 0.05; and interaction effect *F*_1,33_ = 3.15, *P* < 0.05), LH (group effect *F*_1,33_ = 5, *P* < 0.05), and RH (group effect *F*_1,33_ = 7.53, *P* < 0.05). Post hoc analysis of the RF values showed that MCAO animals had increased stance duration postoperative day 6 (*P* < 0.05) and 42 (*P* < 0.05) (Figure 4(a)). However, when we compared the contralateral versus ipsilateral sides of the animals, we did not find significant differences.

The swing duration (average time in seconds in which the paw is not in contact with the glass plate) was significantly increased only in the LF (group effect *F*_1,33_ = 6.51, *P* < 0.05) (Figure 4(b)). As we found that the run speed slightly decreased and stance duration increased in MCAO animals, the total run duration was consequently significantly longer (group effect *F*_1,33_ = 5.28, *P* < 0.05), and the swing speed was also greater for all four paws of MCAO rats (Table 1).

Taken together, our data from gait analysis during the 42-day followup after MCAO demonstrate that ischemia affected both contralateral and ipsilateral paws and also affected both forepaws and hindpaws. These results confirmed that the deficit was stable during the followup.

### 3.3. Comparative Paw Statistics

Consistent with data in stroke patients [12], we found that MCAO rats presented significantly shorter stride length (distance between successive placements of the same paw during maximal contact) in the LH (group effect *F*_1,33_ = 7.28, *P* < 0.05; and interaction effect *F*_1,33_ = 3.05, *P* < 0.05) at postoperative day 42 (*P* < 0.05) (Figure 4(c)). This was also true for the RF (group effect *F*_1,33_ = 4.85, *P* < 0.05; and interaction effect *F*_1,33_ = 3.24, *P* < 0.05) and for the RH (group effect *F*_1,33_ = 3.06, *P* < 0.05; and interaction effect *F*_1,33_ = 3.05, *P* < 0.05). Post hoc analysis showed that the differences were present at both postoperative day 6 (*P* < 0.05) and day 42 (*P* < 0.05). Statistical analysis showed no significant differences for the LF (group effect *F*_1,33_ = 2.582, *P* = 0.13). 

MCAO rats showed significantly longer step cycles (the time in seconds between two consecutive contacts of the same paw) for the RF (group effect *F*_1,33_ = 9.02, *P* < 0.05), RH (group effect *F*_1,33_ = 9.65, *P* < 0.01), LF (group effect *F*_1,33_ = 8.68, *P* < 0.05), and LH (group effect *F*_1,33_ = 6.57, *P* < 0.05; and interaction time × group effect *F*_1,33_ = 2.91, *P* < 0.05). Detailed post hoc analysis of the LH values showed significant differences at day 6 (*P* < 0.05) and day 42 (*P* < 0.05) after reperfusion (Table 1). However, we did not find significant differences between the contralateral and ipsilateral sides of the animal. 

When we analyzed duty cycles (the percentage of time the paw accounts for the total step cycle of the paw), we found that this only varied in the RF of MCAO animals (interaction effect *F*_1,33_ = 3.91, *P* < 0.05; and time effect *F*_1,33_ = 4.30, *P* < 0.05) at postoperative day 6 (*P* < 0.05) (Figure 4(d)). Moreover, the base of support of the hindpaws significantly increased at postoperative day 42 (time effect *F*_1,33_ = 5.62, *P* < 0.01; and interaction time × group effect *F*_1,33_ = 3.78, *P* < 0.05) (Figure 3(b)). As the base of support is the average width between either the front paws or the hindpaws; MCAO animals showed wider steps with their hindpaws, most likely as a mechanism to compensate for their unsteady gait.

### 3.4. Interlimb Coordination or Individual Footprint Parameters Were Not Affected by Ischemia

No differences between the sham-operated and MCAO group were detected when interlimb coordination parameters were analyzed. The overall interlimb coordination during gait (known as gait regularity index of step sequence) was not affected, and all values remained above 95% for both groups during the followup. Another parameter to assess interlimb coordination is phase dispersion (the temporal relationship between placements of two paws within a step cycle). Phase dispersion between diagonal limb pairs (i.e., LF to RH and RF to LH) showed no differences during the 42-day followup after MCAO (Table 1). Interlimb coordination in ipsilateral pairs (i.e., LF to LH and RF to RH) as well as between front paws was not altered by MCAO (data not shown). In line with previous results in phase dispersion, we did not observe irregularities in the overall step sequence, but we found the alternative pattern AB in majority of sham-operated and MCAO rats (data not shown).

Unlike the findings reported by Wang et al. [6], we did not find significant differences between sham-operated and MCAO rats when the maximum paw contact area, maximum intensity, print area, print width, print length, and toe spread were analyzed. However, a strong time effect was observed during the followup of both MCAO and sham-operated animals (Table 1), which led us to believe that body weight had influenced all these gait parameters.

### 3.5. Body Weight Affects CatWalk Parameters

Animals were 358 ± 10 g at the beginning of the study. Due to the surgical procedures, all the animals lost some body weight after their operation. Particularly, the MCAO group showed severe loss of weight (11 ± 5%) during the first week after ischemia, but this was followed by progressive weight gain during the rest of the followup (time effect *F*_1,33_ = 107.9, *P* < 0.001; and interaction effect *F*_1,33_ = 4.42, *P* < 0.01). 

**Figure 5:**
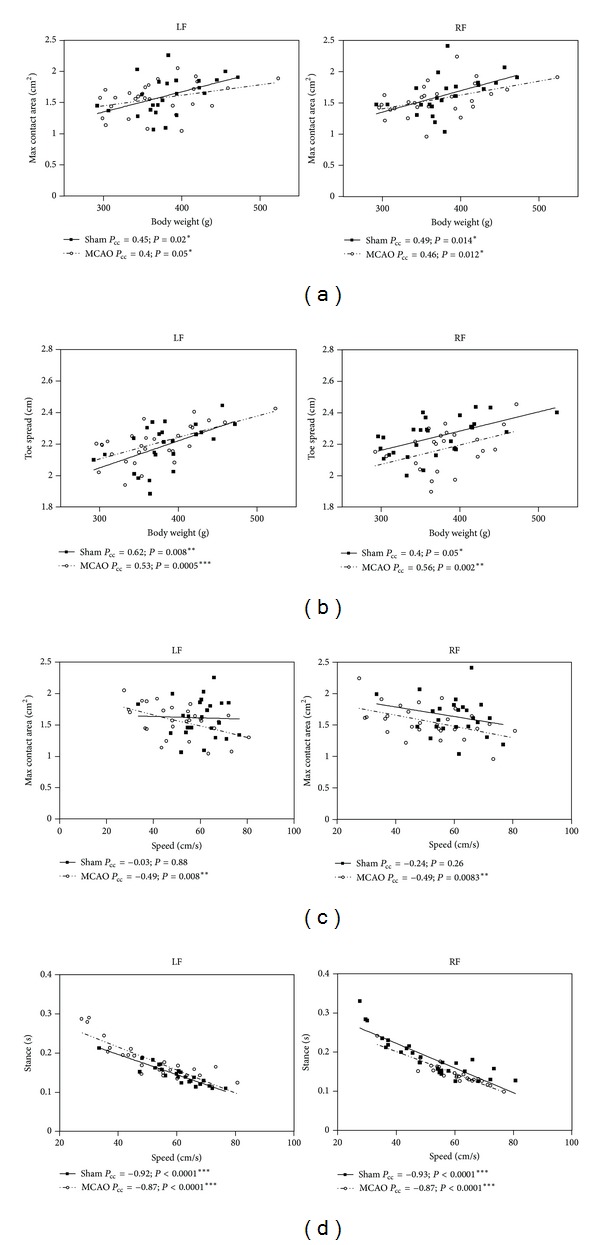
Gait parameter correlations. Scatter plots showing the correlations between gait parameters and body weight ((a), (b)) and locomotor speed ((c), (d)) of the left and right forepaws. Linear regression lines were plotted in each group (solid lines for sham-operated and dotted lines for MCAO rats). The values of Pearson's Products Moment Correlations Coefficients (*P*_cc_) and *P*-values are shown for each group. Maximum contact area (a) and toe spread (b) showed positive correlation with body weight. By contrast, the maximum contact area (c) and stance (d) showed negative correlations with locomotor speed. Values for the left and right hindpaws followed the same correlation patterns (data not shown).

CatWalk uses the light reflected by the paws as they contact the glass of the apparatus. As explained above, some gait parameters showed a characteristic feature, in which the values increased during the followup, and this seemed to be intimately related to weight recovery. Therefore, to assess whether body weight could affect CatWalk parameters, we conducted Pearson's correlation analysis (Figure 5). As described previously [13, 14], we found a strong negative correlation between the body weight loss and the severity of the lesion (sham *P*_cc_ = −0.89, *P* < 0.05; MCAO *P*_cc_ = −0.85, *P* < 0.05), whereby those animals with larger infarcts suffered more acute weight loss. Conversely, body weight did not affect run duration, speed, or stride length (Table 2). In addition, we also found that body weight was positively influencing swing speed and the maximum intensity of sham-operated animals, but weight did not influence the values for forepaws (Table 2) or hindpaws (data not shown) of MCAO rats. This was probably due to the differences in the weight recovery rates of both groups within the first week after ischemia. Individual paw parameters such as maximum paw contact area (Figure 5(a)), paw print area, paw print width, and toe spread (Figure 5(b)) from both sham and MCAO groups were also affected by body weight for the forepaws (Table 2) and the same was true for hindpaws (data not shown). Therefore, we here showed that some of the parameters measured by the CatWalk system were interdependent on weight gain in both the fore and hindpaws. Thus, in cases where the body weight of the animals is affected by the surgical procedure (i.e., cerebral ischemia) the results can become biased.

### 3.6. Temporal Parameters Can Also Directly Affect Other Gait Parameters

The motivation of the animals to walk towards the goal box is very important for the generation of reliable data. Unmotivated animals had difficulties completing their runs, which affect their speed and consequently cadence, and also other temporal parameters. Interestingly, speed showed strong negative correlation with maximum contact area in MCAO animals (Figure 5(c)), indicating that when there is a decrease in walking speed due to infarct mediated impairment, rats increased the contact area of the paw with the walking surface. Also, stride length and stance (Figure 5(d)) showed an intimate interdependency with speed in the fore- and hindpaws of both sham-operated and MCAO rats. When the speed increased the animals had longer stride lengths and decreased stance duration. There was no correlation between temporal parameters and individual paw or interlimb coordination (Table 2). As cadence and speed showed very similar behaviors, when we correlated cadence with gait parameters, we observed that cadence was influencing the same parameters that speed did (data not shown).

## 4. Discussion

The CatWalk system was initially designed to measure gait in models of spinal cord injury, neuropathic pain, and peripheral nerve injury [15]. In stroke animals, previous studies have shown transient, variable, and/or minor gait impairment [6–9], which may be partly due to the different models and time points selected for testing. In the present study, rats subjected to transient MCAO showed a severe and long-lasting impairment when sensitive sensorimotor tests were applied (e.g., cylinder test, sticky label test, and Montoya's staircase). In contrast, only minor impairment was observed in gait after MCAO, which is consistent with behavioral observation—gross locomotion is seemingly normal a few weeks after MCAO. 

### 4.1. Body Weight Contributes to a Variety of Gait Parameters

The body weight of the animal is considered as a possible confounder of gait data [16, 17], and measures such as intensity and maximum contact area are obviously affected by the weight of the animals. Thus, to assess whether body weight was affecting gait parameters, we measured the animal weight every day after ischemia induction. Consistent with the findings by Koopmans et al. [17], we hence found an interdependency of body weight with contact area, print area, and print width. Particularly in the MCAO model, this interdependency may have an impact on gait results, since there is a severe initial loss of body weight after surgery, related to lesion size and neurological impairment [14, 18, 19]. More importantly, this might also be relevant in other models involving long-term followup with progressive gain of body weight. 

### 4.2. Walking Speed and Motivation Are Key Factors Affecting CatWalk Data

Data acquisition on the CatWalk system depends on the velocity of the animals when walking across the runway. In line with previous studies [6, 20], we showed that both walking speed and cadence were directly associated with stride length and temporal parameters such as stance and swing speed. The CatWalk system allows setting up a maximum speed variation within runs. This value is a compromise between obtaining easy runs but reliable data as well. Based on a pilot study, we here selected a maximum speed variation of 50%. Although the differences in temporal parameters between MCAO animals and sham-operated animals are most likely due to the impairment caused by ischemia, they can also be affected by the speed, which intimately depends on the motivation of the animal to walk towards the goal box. Consequently, these interdependencies may lead to misinterpretation of experimental data.

In addition, as explained above, the motivation of animals to complete the task plays a crucial role in data acquisition. In order to improve the motivation and in turn to prevent stopping and turning around, the runway is connected to a goal box with a cage underneath. However, this did not help and we noted that animals showed reduced motivation to cross the test runway upon repeated testing (habituation). Due to technical reasons, the runs have to be carried out in a dark environment in which the rats are more likely to display exploratory behavior [21]. Thus, it is important to motivate rats properly to complete a run with appropriate speed in a dark and relatively safe environment. Alternative positive motivators to be considered are smell, noise, and food reward [22]. On the other hand, gait impairment may completely disappear when the animals are performing a highly motivated behavior [22]. And lastly, we are not sure whether the other behavioral tests (i.e., cylinder test, sticky label test, and Montoya's staircase) to which the rats were subjected might have interfered with their CatWalk performance.

### 4.3. Impact of Ischemic Damage on Gait Impairment in MCAO Rats

MCAO rats showed a large corticostriatal lesion followed by secondary damage/pathology in the thalamus [23], which may contribute to late impairment. Indeed, delayed worsening in several gait parameters was observed at postoperative day 42. Previously, the impact of cortical and subcortical lesions on gait is suggested to be transient and minor [24]. In line with this, there is evidence that the corticospinal tract does not contribute to overground locomotion, but is required for skilled locomotion such as ladder walking [25–27]. Interestingly, this indicates that in the absence of supraspinal input, the spinal cord is able to generate basic stepping and modulate locomotor activity [28].

Much to our surprise some gait parameters were altered bilaterally in MCAO rats. Having a closer look on previous studies, bilateral impairment (e.g., print area, max area) was also observed by Wang et al. [6]. Unilateral lesions are known to result in impairments of both sides of the body [29]. However, the bilateral gait adjustment we observed here is most likely related to compensation, which may help stabilize the animal during voluntary locomotion [22]. It seems that ischemic rats are partially able to compensate by postural adjustments and shifting body weight to the intact limbs but also by adjusting the gait of the ipsilateral limb according to the contralateral side. The proper gait for terrestrial walking is needed for escape. Thus, bilateral gait adjustment/compensation may be an evolutionary mechanism needed for survival.

At first, gait analysis seemed to be an attractive and feasible approach in MCAO rats. However, a high sample size (15–20 animals/group) is needed to detect a therapeutic effect large enough to notice a gait improvement [9]. Particularly, to observe a treatment effect in our study and by using the stride length as an example, we would only need 8 animals per group to obtain a relatively large effect size (*d* = 1.4 and *α* = 0.05; *β* = 0.08). However, we would need to increase the number of animals per group to *n* = 25–30 to observe what is considered to be a medium size effect (*d* = 0.75). Thus our data suggests that CatWalk can be a helpful complementary tool when testing therapeutic compounds in experimental models of stroke if combined with other tests that are not dependent on weight, speed, or compensatory strategies.

### 4.4. How Similar Is Gait Impairment in Rats versus Humans after Stroke? 

The gait impairments in MCAO animals were subtle, but persistent, and resembled those of patients with stroke such as the decreased cadence and in the increase in the base of support. Both of these alterations in gait have been observed in hemiplegic stroke patients [12, 30]. The general markers of interlimb coordination were unaltered in MCAO animals, which again suggests similarity with the gait of stroke patients, namely, that the central nervous system controls impaired gait by controlling the speed performance within the limits of the available compensatory behavior between affected and unaffected sides [31, 32].

## 5. Conclusions

CatWalk produces an exhaustive number of gait parameters that are potentially useful in the assessment of motor behaviors in MCAO rats. Some parameters are affected by body weight, speed, and motivation, even when a goal box is used, which may confound the data interpretation. In addition, compensatory adjustments develop to stabilize locomotion after a severe ischemic lesion. Although bipedal versus quadrupedal gait impairment after stroke seems to share some similarities, the translational applicability of CatWalk data remains open and further work is needed to explore this issue. 

## Figures and Tables

**Table 1:** CatWalk gait parameter statistics.

	MCAO		Time		Interaction	
	*F* value	*P* value	*F* value	*P* value	*F* value	*P* value
*Temporal parameters *						
Cadence	9.611	0.010*	3.173	0.037*	2.012	0.131
Stance duration						
RF	8.692	0.013*	2.917	0.048*	3.015	0.043*
RH	7.538	0.019*	2.553	0.072	2.268	0.098
LF	6.951	0.023*	2.591	0.069	2.605	0.068
LH	5.004	0.047*	2.615	0.067	2.818	0.054
Swing duration						
RF	2.226	0.163	0.088	0.966	0.229	0.875
RH	3.851	0.075	0.875	0.463	0.707	0.554
LF	6.512	0.026*	2.313	0.094	2.379	0.087
LH	3.805	0.077	1.386	0.264	0.583	0.629
Speed	3.667	0.081	2.259	0.099	2.533	0.073
Run duration	5.28	0.042*	1.521	0.227	2.814	0.054
Swing speed						
RF	5.069	0.045*	0.872	0.465	1.991	0.134
RH	9.524	0.010*	1.43	0.251	2.822	0.053
LF	9.858	0.009**	2.406	0.085	2.966	0.046*
LH	7.993	0.016*	1.6	0.208	3.51	0.026*
*Comparative paws *						
Stride length						
RF	4.855	0.049*	1.079	0.371	3.242	0.034*
RH	7.452	0.019*	0.848	0.477	3.058	0.041*
LF	2.582	0.136	0.436	0.728	1.704	0.185
LH	7.228	0.021*	0.799	0.503	3.05	0.042*
Step cycle						
RF	9.026	0.012*	1.465	0.242	2.146	0.113
RH	9.658	0.01**	2.3	0.095	2.621	0.067
LF	8.684	0.013*	2.375	0.087	2.432	0.082
LH	6.571	0.026*	1.987	0.135	2.914	0.048*
Duty cycle						
RF	4.166	0.066	4.305	0.011*	3.91	0.017*
RH	1.296	0.279	2.794	0.055	1.252	0.306
LF	2.713	0.127	1.936	0.143	0.601	0.618
LH	1.164	0.334	0.197	0.659	1.224	0.312
Base of support						
Forepaw	3.547	0.086	1.319	0.284	1.584	0.211
Hindpaw	3.73	0.079	5.626	0.003**	3.785	0.019*
*Interlimb Coordination *						
Regularity index	0.124	0.730	0.123	0.945	0.146	0.931
Phase dispersion (Diagonal)						
LF → RH	1.842	0.202	0.634	0.598	0.219	0.881
RF → LH	3.84	0.075	0.949	0.428	0.391	0.759
*Individual paw *						
Max contact area						
RF	0.737	0.408	5.338	0.004**	0.186	0.905
RH	0.807	0.388	6.443	<0.001***	0.767	0.520
LF	0.179	0.680	3.397	0.029*	0.185	0.905
LH	2.558	0.138	10.29	<0.0001***	0.515	0.674
Max intensity						
RF	0.377	0.551	6.462	<0.001***	1.786	0.169
RH	2.694	0.129	3.256	0.033*	0.102	0.958
LF	0.3162	0.585	3.479	0.026*	0.883	0.459
LH	0.271	0.612	6.399	<0.001***	0.408	0.748

RF: right forepaw; LF: left forepaw; RH: right hindpaw; LH: left hindpaw.

*P < 0.05.

**P < 0.01.

***P < 0.001.

**Table 2:** Relationships between body weight and speed with gait parameters.

	Body weight					Speed			
	Sham		MCAO			Sham		MCAO	
	*P*_cc_	*P* value	*P*_cc_	*P* value		*P*_cc_	*P* value	*P*_cc_	*P* value
Infarct volume	—	—	−0.85	0.015*	Body weight	−0.01	0.95	−0.02	0.90
Run duration					Stride length				
LF	−0.04	0.83	0.08	0.68	LF	0.86	<0.0001***	0.77	<0.0001***
RF	−0.05	0.84	0.07	0.65	RF	0.73	<0.0001***	0.75	<0.0001***
Swing speed					Stance				
LF	0.50	0.012*	0.09	0.62	LF	−0.92	<0.0001***	−0.87	<0.0001***
RF	0.42	0.042*	0.10	0.61	RF	−0.93	<0.0001***	−0.87	<0.0001***
Max intesity					Max contact area				
LF	0.42	0.042*	0.20	0.28	LF	−0.03	0.88	−0.49	0.0086**
RF	0.40	0.049*	0.23	0.23	RF	−0.24	0.26	−0.49	0.0083**
Max contact area					Max intensity				
LF	0.45	0.02*	0.40	0.05*	LF	0.18	0.40	−0.33	0.09
RF	0.49	0.014*	0.46	0.012*	RF	−0.19	0.36	−0.20	0.30
Print width					Print width				
LF	0.53	0.007**	0.54	0.003**	LF	0.33	0.12	0.11	0.59
RF	0.38	0.07	0.58	0.0013**	RF	0.05	0.80	0.10	0.63
Toe spread					Toe spread				
LF	0.62	0.008**	0.53	0.0005***	LF	0.18	0.40	0.11	0.59
RF	0.40	0.05*	0.56	0.002**	RF	0.31	0.14	0.27	0.17
Stride length					Base of support				
LF	0.10	0.62	0.11	0.56	Forelimb	0.08	0.71	0.15	0.46
RF	0.18	0.40	0.21	0.29	Hindlimb	0.08	0.70	0.28	0.15

RF: right forepaw; LF: left forepaw.

*P < 0.05.

**P < 0.01.

***P < 0.001.
